# Willingness to Receive SARS-CoV-2 Vaccination and Associated Factors among Chinese Adults: A Cross Sectional Survey

**DOI:** 10.3390/ijerph18041993

**Published:** 2021-02-18

**Authors:** Lin Gan, Yan Chen, Peipei Hu, Dawei Wu, Yajuan Zhu, Jinlin Tan, Yufen Li, Dingmei Zhang

**Affiliations:** 1 Department of Epidemiology, School of Public Health, Sun Yat-Sen University, Guangzhou 510080, Guangdong, China; ganlin3@mail2.sysu.edu.cn (L.G.); hupp3@mail2.sysu.edu.cn (P.H.); wudawei@mail.sysu.edu.cn (D.W.); zhuyj58@mail2.sysu.edu.cn (Y.Z.); tanjlin3@mail2.sysu.edu.cn (J.T.); liyf276@mail2.sysu.edu.cn (Y.L.); 2 Medical College, Shaoguan University, Shaoguan 512000, Guangdong, China; vickycc_chen@163.com

**Keywords:** COVID-19, SARS-CoV-2 vaccine, vaccination, willingness, China

## Abstract

Vaccination is a key strategy to prevent the pandemic caused by the coronavirus disease 2019 (COVID-19). This study aims to investigate the willingness of Chinese adults to be vaccinated against COVID-19 and further explore the factors that may affect their willingness. We used a self-design anonymous questionnaire to conduct an online survey via the Sojump. A total of 1009 valid questionnaires were analyzed. The age of the participants ranged from 18 to 74. Among them, 609 (60.4%, 95%CI: 57.4–63.4%) were willing to receive the severe acute respiratory syndrome coronavirus 2 (SARS-CoV-2) vaccine. Logistic regression analysis results showed that the age of 30–49 (OR = 2.042, 95%CI: 1.098–3.799), universities and colleges education (OR = 1.873, 95% CI = 1.016–3.451), master degree or above education (OR = 1.885, 95%CI = 1.367–2.599), previous influenza vaccination history (OR = 2.176, 95%CI: 1.474–3.211), trust in the effectiveness of the vaccine (OR = 6.419, 95%CI: 3.717–11.086), and close attention to the latest news of the vaccine (OR = 1.601, 95%CI: 1.046–2.449) were facilitative factors that affected their willingness to be vaccinated. More than half of the adults in China would be willing to receive a SARS-CoV-2 vaccine. Middle-aged people with higher education, those who had been vaccinated against influenza, and those who believed that COVID-19 vaccine was effective and paid close attention to it were more willing to be vaccinated. Our findings can provide reference for the implementation of vaccination and the prevention of COVID-19 in China. More studies are needed after the vaccine is launched.

## 1. Introduction

The outbreak of the coronavirus disease 2019 (COVID-19) caused by a novel coronavirus, severe acute respiratory syndrome coronavirus 2 (SARS-CoV-2), has rapidly evolved to be a pandemic, thereby posing a major public health threat worldwide [1]. At present, the pandemic caused by COVID-19 has been basically under control in China [2]. However, the global pandemic is still spreading [3,4]. Vaccination is probably the most effective approach to prevent and control COVID-19 in the future. At present, various SARS-CoV-2 vaccines with different characteristics, such as inactivated vaccine, subunit vaccine, DNA vaccine and mRNA vaccine, are under development at different stages [5,6]. Several vaccines have entered Phase III clinical trials, and the safety and effectiveness of these vaccines will be tested via a large-scale vaccination of subjects [7].

In July 2020, inactivated SARS-CoV-2 vaccine was approved for emergency use in special populations in China under the condition of lack of sufficient Phase III clinical trial data about the safety and effectiveness of the vaccines. More than 24 million doses of the SARS-CoV-2 vaccine had been administered in China till January 31, 2021 [8]. The two vaccines currently in use in China are the China National Biotec Group (CNBG) COVID-19 vaccine and the CoronaVac vaccine developed by China’s Sinovac Biotech Ltd. The willingness of people to receive SARS-CoV-2 vaccine will be necessary for the implementation of vaccination and the prevention of COVID-19 in China. Thus, exploring the factors that affect the willingness to vaccinate and reasons for the reluctance and hesitancy to receive a SARS-CoV-2 vaccine will be essential to inform ethical and scientific decisions for the launch of SARS-CoV-2 vaccines in China in the near future. A recent research explored the willingness of young students in China to be vaccinated against COVID-19, indicating that over 60% students were willing to be vaccinated. Low socioeconomic status and female gender were facilitative factors that affected the willingness to be vaccinated [9]. However, the population of this study included students who were relatively less likely to be infected and could not represent the whole age population. A study in the United States found that 69% of the participants were willing to receive a SARS-CoV-2 vaccine. Participants who were more likely to be infected were more likely to receive the vaccine [10]. In another survey study of the US people, vaccine-related attributes were associated with their willingness to receive vaccination. Vaccine efficacy was associated with the willingness to receive a vaccine [11]. Another study indicated that education level could affect the willingness to be vaccinated against COVID-19. People with a bachelor or higher degree were more likely to receive a SARS-CoV-2 vaccine [12]. A previous study on willingness to be vaccinated against influenza among Chinese parents showed that higher level of knowledge about influenza was positively correlated with the willingness to vaccinate [13].

At present, survey on the willingness to receive the SARS-CoV-2 vaccine of the adults in China are scarce. Accordingly, we conducted an online survey via the Sojump from 23 October to 10 November 2020, aiming to investigate the willingness to be vaccinated against COVID-19 among Chinese people and factors influencing the willingness. Our findings could provide reference for the implement of SARS-CoV-2 vaccination in China.

## 2. Materials and Methods

### 2.1. Study Design and Population

The convenience sampling method was conducted in this cross-sectional study. We used a self-design anonymous questionnaire to conduct an online survey from 23 October to 10 November 2020. Participants were recruited via the Sojump (https://www.wjx.cn/ accessed on 3 February 2021), which is the most commonly used online survey tool in China. We published the questionnaire on the Internet and recruit participants by sharing links or QR codes via WeChat (the largest social platform in China). We used the formula for sample size in the cross-sectional study to determine our study group, n = Z_α_^2^ × proportion (1−proportion)/precision^2^. In the formula, α = 0.05, Z_α_ = 1.96, and the precision is 0.05. The proportion is 64.01%, which is the rate of people who are willing to be vaccinated against COVID-19 according to a previous study in China [8]. After calculation, the minimum sample size was 354. One thousand and eleven questionnaires were collected during the investigation. After excluding two invalid questionnaires with unreasonable birthday information, a total of 1009 valid questionnaires were analyzed in this study eventually.

### 2.2. Questionnaires

The questionnaire comprised four parts. The first part collected demographic information (e.g., age, gender, residence, degree of education, history of disease, and occupation) and socio-economic status (e.g., household income). This part also enquired whether the participant had been vaccinated against influenza. The second part investigated the knowledge of the participant about SARS-CoV-2 and COVID-19 (e.g., the route of transmission, quarantine days, symptoms, clinical classification, and preventive measure). We set up 9 questions to answer “yes” or “no” in this part to explore the knowledge level. When a question was answered correctly, the participant received 1 point. Otherwise, it would not be scored. The sum of the points of the 9 questions was recorded as the total score. We used the lower quartile as a cut-off to distinguish different score groups. Higher scores suggested a better understanding on COVID-19 and SARS-CoV-2. In the third part, the participants were asked about their willingness to be vaccinated, that is, whether they would be vaccinated against COVID-19 and reasons for their reluctance or hesitation. In the last part, we set up 10 questions to investigate the hygiene habits of the participants. When the respondent choose option answers with better hygiene habit, he/she will receive 1 point. Otherwise, it would not be scored. The sum of the points of the 10 questions was recorded as the total score. Participants with higher scores had better hygiene habits. Scores below 5 points (lower quartile) are considered that the respondent does not have a good hygiene habit. The validation of this questionnaire was mainly examined and approved by professors and relevant professionals before being distributed. The reliability of this questionnaire was examined by Kuder–Richardson test (KR-20 = 0.411 for knowledge scores; KR-20 = 0.509 for hygiene habits scores). The scores for KR-20 range from 0 to 1, where 0 is no reliability and 1 is perfect reliability. The closer the score is to 1, the more reliable the test. We used this questionnaire to explore the willingness of the participants to receive the SARS-CoV-2 vaccine and we also explored the factors, such as the sociodemographic characteristics of the participants, their personal hygiene habits, and their knowledge about SARS-CoV-2 and COVID-19, that might affect the willingness to be vaccinated.

### 2.3. Statistical Analysis

Information was collected from Sojump. All data were analyzed by SPSS statistics 25.0 software (SPSS Inc., Chicago, IL, USA). Categorical variables were compared by using chi-squared test or Fisher’s exact test. The potential factors that influence vaccination willingness, such as age, occupations, socio-economic status, knowledge about SARS-CoV-2 and COVID-19, and personal hygiene habits were initially assessed using univariate logistic regression analysis. Factors with *p* < 0.1 [14,15] in univariate logistic regression analysis were included in the final multiple logistic regression analysis, and *p* < 0.05 was set as a significant difference. Odds ratios (ORs) and 95% confidence intervals (CIs) were used to estimate associations.

### 2.4. Quality Control

To ensure the reliability of the results, the participants in this study were recruited without any financial remuneration. The questionnaires with incorrect age information were considered invalid questionnaires and were thus excluded.

## 3. Results

### 3.1. Sociodemographic Characteristics

A total of 1009 valid questionnaires were analyzed in this study. Among the participants, 609 (60.4%) were willing to receive SARS-CoV-2 vaccine, 72 (7.1%) were not willing to receive the vaccine, and 328 (32.5%) were unsure. Most participants were female (62.1%) and lived in urban area (89.7%). The age of the participants ranged from 18 to 74, with the median age 30 years old. A total of 46.9% of participants aged 18–29, 45.7% aged 30–49, and 7.4% aged 50 and older. Among the participants, only 93 (9.2%) had underlying diseases before admission. A total of 387 participants had children. Among them, 200 (51.7%) were willing to vaccinate their children, 42 (10.9%) were unwilling to vaccinate their children, and 145 (37.4%) were uncertain. More than half of the participants reported a monthly household income of less than $1245 (59.3%), and the income of the vast majority of participants has remained unchanged (50.8%) or decreased (46.3%) after the COVID-19 outbreak. Most of the participants have a college degree or above (89.1%), no respiratory diseases experience in the past year (59.1%), and no previous history of influenza vaccination (82.2%). Among the participants, students (26.8%), hospital and Centers for Disease Control and Prevention staff (19.5%), company staff (13.7%), and teachers (11.6%) account for a large proportion. Education level, occupations, and influenza vaccination history showed statistically significant differences among those who were willing to receive the vaccine, unwilling to receive the vaccine, and unsure to be vaccinated (Table 1).

### 3.2. Knowledge about SARS-CoV-2 and COVID-19

We set up a total of 9 knowledge questions about SARS-CoV-2 and COVID-19 (Table 2). The first question, which was about the mode of communication of SARS-CoV-2, had the lowest correct response rate at 70.7%. A total of 169 (16.7%) participants did not choose “contact communication,” indicating that some participants ignored this mode of communication. A total of 115 participants (11.4%) did not know that COVID-19 patients would have other symptoms besides fever. Other questions about COVID-19 had a correct response rate of over 90%. The correct response rates of the three questions differed significantly between the participants who were willing and those who were not willing or unsure to receive a SARS-CoV-2 vaccine. These questions were the mode of communication of SARS-COV-2 (*p* = 0.027), participants who have been exposed to asymptomatic infections of SARS-CoV-2 may be infected (*p* = 0.046), and different clinical types of COVID-19 patients (*p* = 0.038). Total knowledge score has statistical difference for participants who were willing (median = 9, IQR: 8-9) and those who were unwilling or unsure (median = 8, IQR: 8–9) to receive the vaccine (*p* = 0.007).

### 3.3. Personal Hygiene Habits of Participants

Ten questions about personal hygiene habits in the last month were raised in this part. All of the choices, such as washing your hands immediately after returning home (*p* < 0.001), washing your hands with soap or hand sanitizer (*p* < 0.001), and sharing towels with your family (*p* = 0.012) showed statistically significant differences between the participants who were willing and those who were not willing or unsure to receive the SARS-CoV-2 vaccine (Table 3). The overall hygiene habit score for the participants who were willing to receive the vaccine (median = 7, IQR: 6–9) was significantly different (*p* = 0.015) from those who were unsure or unwilling (median = 7, IQR: 6–8).

### 3.4. Factors Associated with the Willingness to Vaccinate against COVID-19

We conducted logistic regression to evaluate the association among sociodemographic characteristics, knowledge about SARS-CoV-2 and COVID-19, personal hygiene habits, and willingness to receive SARS-CoV-2 vaccine. The results of univariable logistic regression analysis showed that six factors, including education level (*p* < 0.001), occupations (*p* = 0.028), previous influenza vaccination history (*p* < 0.001), trust the effectiveness of the vaccine (*p* < 0.001), pay attention to the latest news of the vaccine (*p* = 0.004), and total knowledge score (*p* = 0.031), were associated with the willingness to receive the vaccine (Table 4). These factors were further put into the multiple logistic regression analysis model, together with the family income factor that probably has an impact according to common sense and other possible confounding factors. Eventually, the results showed that middle-aged people (30–49 years old) were more willing to be vaccinated (OR = 2.042, 95% CI = 1.098–3.799). We also found that compared with participants with high school education or below, those with universities and colleges education (OR = 1.873, 95% CI = 1.016–3.451) and master degree or above (OR = 1.885, 95% CI = 1.367–2.599) were more likely to be vaccinated against COVID-19. Participants who had been vaccinated against influenza in the past were more willing to receive the SARS-CoV-2 vaccine (OR = 2.176, 95% CI = 1.474–3.211). Participants who trust the effectiveness of the vaccine were more willing to be vaccinated (OR = 6.419, 95% CI =3.717–11.086). Compared with participants who did not care much about the SARS-CoV-2 vaccine, those who pay close attention to the latest news of the vaccine were more willing to be vaccinated (OR = 1.601, 95% CI = 1.046–2.449). The model passed the Hosmer and Lemeshow test (χ^2^ = 5.347, df = 8, *p* = 0.720), indicating that this model was a good fit. The independent variables in the model were tested for collinearity. The results showed that the tolerance > 0.1, VIF < 10, and no collinearity among independent variables (Table 5).

### 3.5. Main Reasons for Refusing or Hesitating to Vaccinate

The main reasons for refusing or hesitating to be vaccinated were analyzed from the data of the 400 participants who were unwilling or unsure to receive the vaccination. More than one option was allowed to be chosen by the respondents. The results showed that among the participants who were unwilling to be vaccinated, 66.7% thought that the safety of the vaccine may not be enough; 45.8% prepared to observe the first stage of vaccination before deciding whether to be vaccinated, and 40.3% thought that the probability of COVID-19 epidemic in the places where they live is very small, so there is no need to be vaccinated. The main reasons for the participants’ hesitation were also the three aforementioned reasons (Table 6).

## 4. Discussion

SARS-CoV-2 vaccine is the most potential effective way to deal with the pandemic at present. This study investigated the willingness of Chinese participants to receive the SARS-CoV-2 vaccine and the factors that affect their willingness. A total of 60.4% of adults in China would be willing to receive a SARS-CoV-2 vaccine. This result is similar to a previous study, where 64.01% of Chinese participants indicated their willingness to receive the SARS-CoV-2 vaccine [9].

Participants with various sociodemographic characteristics showed different willingness to be vaccinated. Middle-aged people (30–49 years old) were more willing to be vaccinated. Comparing with students in a previous study, these people were at greater risk of infection [9]. Those with higher education level (e.g., master degree or above) were more willing to be vaccinated. Perhaps these participants had a better understanding of COVID-19 and its vaccine than those with lower educational background. A previous study on influenza vaccination in Shanghai obtained the same result [16]. This result also showed the importance of publicity and education among participants with low education level. We found that teachers, staffs of hospitals and CDC, and students were more willing to be vaccinated against COVID-19. This situation may be attributed to the particularity of their work. Participants who had been vaccinated against influenza in the past were more willing to receive the SARS-CoV-2 vaccine, thereby suggesting that a secure vaccination experience of the viral vaccine was sufficient to make participants confident about being vaccinated, which was consistent with a previous research [17].

With regard to knowledge about SARS-CoV-2 and COVID-19, the rate of correct responses to the 9 questions were all over 70%, thereby showing that most participants had a satisfactory understanding of SARS-CoV-2 and COVID-19. This circumstance is probably attributed to the propaganda of Chinese government and the popularization of media dissemination on the prevention and control of COVID-19. Some participants did not have a good understanding of contact transmission, and they may ignore this mode of transmission and infection. Participants with higher knowledge score were more willing to be vaccinated, suggesting that participants who are more knowledgeable about COVID-19 were more assured of the vaccination.

We also found that people who trust the effectiveness of the vaccine were more willing to be vaccinated. They believe that vaccination could prevent SARS-CoV-2 infection effectively. Additionally, people who pay more attention to the latest news of the vaccine were more willing to be vaccinated. These people knew more about COVID-19 vaccine than others.

Furthermore, the results indicated that the “SARS-CoV-2 vaccine may not be safe enough (62.5%)” and “preparing to observe the first stage of vaccination before deciding whether to vaccinate (56.5%)” were the main reasons for reluctance or hesitance to be vaccinated. Participants do not trust the new vaccine, so some participants will take a wait-and-see attitude. Negative news reports about vaccines may also affect participants’ willingness to be vaccinated.

Up to now, more than 100 million SARS-CoV-2 vaccines have been given around the world. A survey in Ecuador showed that a very large proportion of individuals (at least 97%) were willing to accept a COVID-19 vaccine [18]. However, a study in the United States in October showed that the willingness to vaccinate was only 53.6% [19]. Our study found that 51.7% of the respondents who had children were willing to vaccinate their children. But in a study in the United States, 65% of caregivers reported that they intend to vaccinate their child against COVID-19 [20]. A study of US adults showed that vaccine-related attributes, political factors, the age and gender of the participants could affect the willingness to vaccinate [11]. Our research found that close attention and trust in SARS-CoV-2 vaccines will also affect the willingness to vaccinate.

Our research aims to investigate the willingness of Chinese adults to receive SARS-CoV-2 vaccination and their associated factors. This study includes a large sample size and explores a wide range of possible influence factors. The main limitation of our study is that we recruited a convenience sample via the Sojump, which can cause selection bias. Additionally, our study assessed the willingness to be vaccinated under the condition that the SARS-CoV-2 vaccine had not yet been marketed, indicating that the results may not truly reflect the willingness to be vaccinated after marketing. Finally, most of our participants were urban residents with higher education level, and the results cannot represent the majority of the population in China. However, the urban population is relatively dense, and the risk of infection is higher than that of the rural population. Nevertheless, our study can still indicate that more than half of the Chinese participants are willing to be vaccinated against COVID-19 at present. Popularizing the SARS-CoV-2 vaccine in the near future is necessary.

## 5. Conclusions

Our finding made a preliminary estimate of the willingness of Chinese adults to be vaccinated against COVID-19 and the factors affecting the willingness, which can be used to provide guidance for the implementation of vaccines in the future. A total of 60.4% (95%CI: 57.4–63.4%) of adults in China would be willing to receive a SARS-CoV-2 vaccine. The main factors to promote vaccination include the age of 30–49, higher education level, previous influenza vaccination history, trust in the effectiveness of the vaccine, and close attention to the latest news of the vaccine. Our results showed that the participants need to receive more comprehensive health education. However, this prediction is only applicable before the wide utilization of the vaccine. More cross-sectional studies in the future are needed to determine the willingness of Chinese people to receive the SARS-CoV-2 vaccine and its influencing factors.

## Figures and Tables

**Table 1:** Sociodemographic characteristics of the participants for surveys of willingness to get a SARS-CoV-2 vaccine in China.

	Total Participants	Willingness to Receive Vaccine			*p*-Value
		Yes	No	Unsure	
	(*n*/%) (*n* = 1009)	(*n*/%) (*n* = 609)	(*n*/%) (*n* = 72)	(*n*/%) (*n* = 328)	
Gender					0.237
Male	382 (37.9)	236 (38.8)	32 (44.4)	114 (34.8)	
Female	627 (62.1)	373 (61.2)	40 (55.6)	214 (65.2)	
Age (years)					
18–29	473 (46.9)	298 (48.9)	33 (45.8)	142 (43.3)	0.377
30–49	461 (45.7)	272 (44.7)	32 (44.4)	157 (47.9)	
50 and older	75 (7.4)	39 (6.4)	7 (9.8)	29 (8.8)	
Urbanicity					
Urban	905 (89.7)	547 (89.8)	68 (94.4)	290 (88.4)	0.309
Rural	104 (10.3)	62 (10.2)	4 (5.6)	38 (11.6)	
Education level					
High school or below	110 (10.9)	57 (9.4)	5 (6.9)	48 (14.6)	<0.001
Universities and colleges	490 (48.6)	328 (53.9)	31 (43.1)	131 (39.9)	
Master degree or above	409 (40.5)	224 (36.7)	36 (50.0)	149 (45.5)	
Underlying diseases					
Yes	93 (9.2)	53 (8.7)	7 (9.7)	33 (10.1)	0.781
No	916 (90.8)	556 (91.3)	65 (90.3)	295 (89.9)	
Occupations					
Hospital and CDC staffs	197 (19.5)	138 (22.7)	15 (20.8)	44 (13.4)	0.045 ^a^
Service industry	31 (3.1)	15 (2.5)	3 (4.2)	13 (4.0)	
Staffs of government and public institutions	74 (7.3)	50 (8.2)	5 (6.9)	19 (5.8)	
Company employee	138 (13.7)	84 (13.8)	9 (12.5)	45 (13.7)	
Workers, peasants and small traders	66 (6.5)	35 (5.7)	3 (4.2)	28 (8.5)	
Teachers	117 (11.6)	70 (11.5)	12 (16.7)	35 (10.7)	
Students	270 (26.8)	158 (25.9)	19 (26.4)	93 (28.3)	
Unemployed or retired	34 (3.4)	17 (2.8)	1 (1.4)	16 (4.9)	
Others	82 (8.1)	42 (6.9)	5 (6.9)	35 (10.7)	
Household income (Per-capita monthly income, $)					
Less than 778	385 (38.2)	226 (37.1)	23 (31.9)	136 (41.4)	0.558
778 to 1245	213 (21.1)	138 (22.7)	15 (20.8)	60 (18.3)	
1245 to 1867	162 (16.0)	97 (15.9)	12 (16.7)	53 (16.2)	
More than 1867	249 (24.7)	148 (24.3)	22 (30.6)	79 (24.1)	
Income changes					
Get more	29 (2.9)	20 (3.3)	1 (1.4)	8 (2.4)	0.652 ^a^
Get less	467 (46.3)	290 (47.6)	34 (47.2)	143 (43.6)	
No change	513 (50.8)	299 (49.1)	37 (51.4)	177 (54.0)	
Suffered from respiratory diseases in the past year					
Yes	413 (40.9)	247 (40.6)	32 (44.4)	134 (40.9)	0.817
No	596 (59.1)	362 (59.4)	40 (55.6)	194 (59.1)	
History of influenza vaccination					
Yes	180 (17.8)	135 (22.2)	6 (8.3)	39 (11.9)	<0.001
No	829 (82.2)	474 (77.8)	66 (91.7)	289 (88.1)	

Note: *p*-values comparing different groups were from χ^2^ test or Fisher’s exact test. Significance difference: *p* < 0.05. ^a^ Fisher exactly.

**Table 2:** Participants’ knowledge about SARS-COV-2 and COVID-19.

Questions	Correct Response (*n*/%)	Willingness to Receive Vaccine		*p*-Value
		Yes (*n*/%) (*n* = 609)	No and Unsure (*n*/%) (*n* = 400)	
1. The mode of communication of SARS-COV-2.	713 (70.7)	446 (73.2)	267 (66.8)	0.027
2. People who have been exposed to asymptomatic infections of SARS-CoV-2 may be infected.	981 (97.2)	587 (96.4)	394 (98.5)	0.046
3. People who have been in close contact with COVID-19 patients need to be quarantined for 14 days	941 (93.3)	570 (93.6)	371 (92.8)	0.600
4. COVID-19 patients could have other symptoms besides fever.	894 (88.6)	545 (89.5)	349 (87.3)	0.273
5. In addition to invading the lungs, SARS-COV-2 could also affect other organs.	957 (94.8)	583 (95.7)	374 (93.5)	0.117
6. COVID-19 patients can be divided into asymptomatic infected patients, mild patients, ordinary patients, severe patients and critical patients.	930 (92.2)	570 (93.6)	360 (90.0)	0.038
7. Mild cases of COVID-19 may turn into severe cases.	990 (98.1)	599 (98.4)	391 (97.8)	0.487
8. Washing hands frequently can prevent SARS-COV-2 infection.	973 (96.4)	589 (96.7)	384 (96.0)	0.549
9. Cleaning and disinfecting common or virus-contaminated products can reduce SARS-COV-2 infection.	941 (93.3)	572 (93.9)	369 (92.3)	0.299

Note: *p*-values comparing different groups were from χ² test. Significance difference: *p* < 0.05.

**Table 3:** Personal hygiene habits of participants (the recent month).

Hygiene Habits	Total (*n*/%)	Willingness to Receive Vaccine		*p*-Value
		Yes (*n*/%) (*n* = 609)	No and Unsure (*n*/%) (*n* = 400)	
1. Wash your hands immediately after returning home.				
Yes	890 (88.2)	509 (83.6)	381 (95.2)	<0.001
No	119 (11.8)	100 (16.4)	19 (4.8)	
2. Wash hands with soap or hand sanitizer.				
Yes	837 (83.0)	479 (78.7)	358 (89.5)	<0.001
No	172 (17.0)	130 (21.3)	42 (10.5)	
3. Share towels with your family.				
Yes	131 (13.0)	66 (10.8)	65 (16.3)	0.012
No	878 (87.0)	543 (89.2)	335 (83.7)	
4. Share tableware with your family.				
Yes	665 (65.9)	410 (67.3)	255 (63.7)	0.241
No	344 (34.1)	199 (32.7)	145 (36.3)	
5. Cover your nose and mouth with paper towels or elbow when sneezing or coughing.				
Yes	956 (94.7)	572 (93.9)	384 (96.0)	0.148
No	53 (5.3)	37 (6.1)	16 (4.0)	
6. The living or working environment usually opens the window for ventilation.				
Yes	973 (96.4)	582 (95.6)	391 (97.7)	0.067
No	36 (3.6)	27 (4.4)	9 (2.3)	
7. Indoor ventilation frequency of living or working environment.				
Three times a day or more	474 (47.0)	288 (47.3)	186 (46.5)	0.517
1–2 times a day	331 (32.8)	205 (33.7)	126 (31.5)	
From time to time, occasionally	168 (16.6)	98 (16.1)	70 (17.5)	
Not at all	36 (3.6)	18 (2.9)	18 (4.5)	
8. Disinfect living or working environment with disinfectant.				
Yes	682 (67.6)	409 (67.2)	273 (68.2)	0.717
No	327 (32.4)	200 (32.8)	127 (31.8)	
9. After the domestic epidemic is alleviated, you will still wear masks in indoor spaces such as elevators.				
Yes	657 (65.1)	410 (67.3)	247 (61.7)	0.069
No	352 (34.9)	199 (32.7)	153 (38.3)	
10. The frequency of changing the mask you wear.				
Every half day or every day.	590 (58.5)	358 (58.8)	232 (58.0)	0.692
Every 2~3 days	349 (34.6)	206 (33.8)	143 (35.7)	
Every week	70 (6.9)	45 (7.4)	25 (6.3)	

Note: *p*-values comparing different groups were from χ² test. Significance difference: *p* < 0.05.

**Table 4:** Univariable logistic regression analysis of influencing factors for willingness to vaccinate against COVID-19.

Predictive Variables	OR (95% CI)	*p*-Value
Gender		0.471
Male	Reference	
Female	0.908 (0.700–1.179)	
Age (years)		0.142
18–29	Reference	
30–49	1.572 (0.963–2.566)	
50 and older	1.328 (0.814–2.167)	
Urbanicity		0.870
Urban	Reference	
Rural	0.966 (0.639–1.461)	
Education level		<0.001
High school or below	Reference	
Universities and colleges	0.888 (0.583–1.354)	
Master degree or above	1.672 (1.275–2.192)	
Underlying diseases		0.524
Yes	1.155 (0.742–1.797)	
No	Reference	
Occupations		0.028
Hospital and CDC staffs	0.952 (0.428–2.119)	
Service industry	2.228 (1.312–3.783)	
Staffs of government and public institutions	0.893 (0.391–2.041)	
Company employee	1.984 (1.034–3.806)	
Workers, peasants and small traders	1.481 (0.853–2.572)	
Teachers	1.075 (0.562–2.058)	
Students	1.418 (0.803–2.506)	
Unemployed or retired	Reference	
Others	1.344 (0.818–2.206)	
Household income (Per-capita income, $)		0.515
Less than 778	Reference	
778 to 1245	0.970 (0.701–1.342)	
1245 to 1867	1.256 (0.860–1.833)	
More than 1867	1.018 (0.680–1.524)	
Income changes		0.301
Get more	0.853 (0.660–1.102)	
Get less	1.356 (0.604–3.045)	
No change	Reference	
Suffered from respiratory diseases in the past year		0.766
Yes	0.962 (0.744–1.243)	
No	Reference	
History of influenza vaccination		<0.001
Yes	2.247 (1.561–3.234)	
No	Reference	
Trust the effectiveness of the vaccine		<0.001
Yes	6.587 (3.898–11.131)	
No	Reference	
Pay attention to the latest news of the vaccine		0.004
Yes	1.743 (1.195–2.542)	
No	Reference	
Total knowledge score group		0.031
7 and below	Reference	
8 and above	1.450 (1.035–2.031)	
Total hygiene habits score group		0.108
5 and below	Reference	
6 and above	1.311 (0.942–1.824)	

Note: Significance difference: *p* < 0.1.

**Table 5:** Multiple logistic regression analysis of influencing factors for willingness to vaccinate against COVID-19.

Predictive Variables	OR (95% CI)	*p*-Value
Age (years)		0.050
18–29	Reference	
30–49	2.042 (1.098–3.799)	
50 and older	1.385 (0.791–2.426)	
Education level		0.001
High school or below	Reference	
Universities and colleges	1.873 (1.016–3.451)	
Master degree or above	1.885 (1.367–2.599)	
History of influenza vaccination		<0.001
Yes	2.176 (1.474–3.211)	
No	Reference	
Trust the effectiveness of the vaccine		<0.001
Yes	6.419 (3.717–11.086)	
No	Reference	
Pay attention to the latest news of the vaccine		0.030
Yes	1.601 (1.046–2.449)	
No	Reference	

Note: Significance difference: *p* < 0.05.

**Table 6:** Reasons for refusing or hesitating to vaccinate against COVID-19 (multi-option).

Reasons	Unwilling (*n* = 72)		Unsure (*n* = 328)	
	*n*	%	*n*	%
SARS-CoV-2 vaccine may not be safe enough	48	66.7	202	61.6
Prepare to observe the first stage of vaccination before deciding whether to vaccinate	33	45.8	193	58.8
The possibility of epidemic in the living area is small	29	40.3	138	42.1
Vaccination is not free or too expensive	10	13.9	100	30.5
The preventive effect of SARS-CoV-2 vaccine may not be enough	25	34.7	74	22.6
In good health, the probability of suffering from COVID-19 is small	21	29.2	52	15.9
Family and friends around are not ready to get vaccinated, so they do not get vaccinated either	7	9.7	19	5.8

## Data Availability

The data presented in this study are available on request from the corresponding author. The data are not publicly available due to the privacy reasons.
